# B cells in acquired aplastic anemia and the exploratory role of rituximab: considerations in pediatric and female patients

**DOI:** 10.3389/fimmu.2026.1737771

**Published:** 2026-01-23

**Authors:** Liangwu Pan, Jianren Lin, Xiaobo Zhou, Yanghui Zeng, Chuanming Huang, Ying Fu

**Affiliations:** Department of Pediatric Hematological Oncology, Shunde Women and Children’s Hospital of Guangdong Medical University, Foshan, China

**Keywords:** aplastic anemia, B cell activation, female, pediatric, rituximab

## Abstract

Aplastic anemia (AA) is a severe hematological disorder characterized by bone marrow failure and peripheral pancytopenia. Immune dysregulation plays a central role in its pathogenesis. Recent studies highlight the critical involvement of activated B cells in the immunopathology of AA, influencing both disease onset and progression. Rituximab, a monoclonal antibody targeting CD20, demonstrates therapeutic potential by modulating B cell function. Notably, immune responses and clinical manifestations differ between pediatric and female patients, affecting the efficacy and safety profile of rituximab. This narrative review synthesizes current knowledge on B-cell involvement in AA, summarizes population-specific immunologic features, and appraises the exploratory evidence regarding rituximab use. By integrating mechanistic insights with available clinical observations, we highlight key gaps in the literature and outline priorities for future research to inform more individualized immunomodulatory strategies in AA.

## 1 Introduction

Aplastic anemia (AA) is a severe hematological disease characterized by bone marrow hematopoietic failure and peripheral pancytopenia. Its etiology is complex, involving genetic, environmental, and immune dysregulation factors. In recent years, with advances in immunological research, AA has been widely recognized as an immune-mediated bone marrow failure disease, whose pathological process is closely related to the abnormal activation and functional dysregulation of various immune cells, including T cells and B cells (1, 2). Deeply elucidating the immune pathogenesis of AA is of great clinical significance for developing targeted immunomodulatory therapies and improving patient prognosis.

Current research generally believes that abnormal T cell activation and its immune attack on hematopoietic stem/progenitor cells are central to the pathogenesis of AA. The decreased number and functional deficiency of regulatory T cells (Tregs), along with abnormalities in the FasL-mediated apoptosis pathway, collectively lead to an imbalance in immune tolerance, thereby triggering autoimmune responses (1). Furthermore, abnormalities in the bone marrow microenvironment, such as endothelial progenitor cell dysfunction and dysregulation of the transforming growth factor-β (TGF-β) signaling pathway, also play important roles in the immunopathology of AA (3, 4). However, although the role of T cells in AA has been extensively studied, the specific mechanisms of B cells, as an important component of the immune system, in the pathogenesis of AA remain unclear, representing a weak link in current research.

Increasing evidence in recent years indicates that B cells also play a key role in the immunopathology of AA. B cells are involved not only in antigen presentation, antibody production, and inflammatory cytokine secretion, but their abnormal activation state is also closely related to disease severity and treatment response (5, 6). For example, abnormal distribution of B cell subsets in the bone marrow of AA patients, with an increased proportion of age-associated B cells (ABCs) positively correlating with interferon-γ levels, suggests their involvement in maintaining an inflammatory state (7). Additionally, pediatric and female AA patients have been reported to exhibit distinct immune phenotype, B cell function, and treatment responses (8–10). These observations raise the possibility that age- and sex-related biological factors may influence B-cell–mediated immune dysregulation in AA, although current evidence remains limited and largely descriptive. Thus, a synthesized summary of B cell–related mechanisms in AA, and their potential variation across clinical subpopulations, is warranted.

Based on these research gaps and the limited clarity regarding B-cell–specific contributions to AA, this article aims to provide a focused narrative synthesis of the immunological mechanisms of B cell activation in AA, with particular attention to features observed in pediatric and female patients. In addition, given growing but still exploratory interest in B-cell–targeted therapy, this review summarizes the available clinical experience with rituximab in AA, recognizing that its use remains off-label and is not endorsed by current treatment guidelines. This narrative review is based on a structured literature search conducted in PubMed, Web of Science, and Embase for studies published between January 2000 and October 2025. Search terms included combinations of “aplas”, “aplastic anemia”, “rituximab”, “B cells”, “immune-mediated cytopenias”, “pediatric”, and “female”. Reference lists of relevant articles were also screened to identify additional studies. Eligible publications included clinical studies, case series, mechanistic investigations, and reviews that addressed B-cell–related immune dysregulation or rituximab use in AA or AA-related immune contexts. Studies focusing solely on unrelated autoimmune disorders were excluded unless they contributed mechanistic insights relevant to B-cell activation or depletion.

## 2 Mechanisms of B cell activation in aplastic anemia

### 2.1 The role of B cells in AA immunopathology

AA is an immune-mediated disorder involving the destruction of hematopoietic stem and progenitor cells (HSPCs). While cytotoxic CD8^+^ T cells are considered the primary effectors (11, 12), B cells also contribute significantly to disease pathogenesis. As antigen-presenting cells (APCs), B cells activate T cells and secrete autoantibodies and inflammatory cytokines, thereby amplifying autoimmune injury.

B cell subset distribution and function are markedly altered in AA patients. The frequency of regulatory B cells (Bregs), particularly IL-10-producing B10 cells, is reduced and inversely correlates with disease severity, indicating a loss of immunoregulatory capacity (13). Single-cell transcriptomic analyses reveal abnormal B cell receptor (BCR) repertoire diversity and clonal expansion in AA bone marrow, suggesting autoantibody-mediated targeting of hematopoietic cells (14). In immune-related pancytopenia (IRP), increased B1 cells and plasma cells, along with detectable autoantibodies, further underscore the role of aberrant B cell activation in certain AA subtypes (15).

Imaging mass cytometry and immunohistochemistry have identified lymphoid-rich “immune hotspots” in AA bone marrow, containing activated T cells, B cells, and plasma cells. These structures highlight the collaborative immune cell interactions that drive hematopoietic destruction (16). Through antigen presentation and autoantibody production, B cells activate autoreactive T cells and promote HSPC damage, thereby advancing AA immunopathology.

Therefore, B cells contribute to AA via multiple mechanisms—antigen presentation, autoantibody secretion, and cytokine release—making them potential therapeutic targets.

### 2.2 B cell activation signaling pathways

Dysregulated B cell activation in AA involves several signaling pathways. The BCR pathway is central to B cell activation and proliferation. In AA, aberrant BCR signaling enhances B cell survival and promotes autoimmune responses (14). Bruton’s tyrosine kinase (BTK), a key mediator of BCR signaling, is upregulated in both B and T cells, contributing to autoreactivity and bone marrow failure (12).

The CD40/CD40L co-stimulatory axis also plays a critical role. CD40L on activated T cells engages CD40 on B cells, driving B cell activation and antibody production (16). Elevated levels of B cell-activating factor (BAFF) and interleukin-6 (IL-6) further support B cell survival, differentiation, and antibody secretion, thereby disrupting immune tolerance (17). Notably, high BAFF levels correlate with graft rejection and AA severity.

Breg dysfunction is another key feature of AA. Bregs maintain immune tolerance via IL-10 secretion. Their numerical and functional deficiency in AA, especially in severe cases, impairs immune regulation and promotes autoimmunity (13). Impaired IL-2/STAT5 signaling may underlie Breg dysfunction and reduced IL-10 production (18).

Thus, AA is characterized by enhanced BCR and CD40/CD40L signaling, elevated BAFF/IL-6, and impaired Breg activity—collectively sustaining abnormal B cell activation and autoimmune injury.

### 2.3 Specificity of B cell activation in pediatric and female patients

Pediatric and female AA patients exhibit distinct B cell activation patterns influenced by developmental and hormonal factors. These population-specific immunological features may contribute to the observed heterogeneity in disease presentation, severity, and treatment response.

In pediatric AA, immune immaturity shapes B cell responses. The frequencies of regulatory and transitional B cells are lower than in adults, potentially potentially permitting unchecked autoimmunity and contributing to immune-mediated bone marrow suppression (10). Metabolic alterations in immune cells—such as dysregulated glycolysis and oxidative phosphorylation—may also affect B cell function and treatment response (19). Advanced immunophenotyping and genomic profiling further demonstrate unique B-cell subset distributions in pediatric patients. Flow cytometry and single-cell RNA sequencing have identified expansions of activated naïve CD19^+^ B cells and double-negative (IgD^-^CD27^-^) B cells, indicating altered B-cell differentiation and effector pathways (20). Elevated expression of activation markers such as CD86 and HLA-DR on B cells in AA reflects a hyperactive immune state consistent with dysregulated B-cell function.

At the transcriptional level, pediatric AA patients display aberrations in BCR signaling and regulatory gene networks. Single-cell transcriptomic analyses have revealed diminished expression of BLIMP-1, a key regulator of B-cell differentiation, which may impair Breg development and reduce immune-tolerogenic capacity (18). Moreover, inadequate activation of the IL-2/STAT5 pathway—critical for Breg survival and IL-10 production—has been associated with reduced regulatory capacity and more pronounced immune dysregulation in pediatric AA (18). Metabolic alterations, including dysregulated glycolysis and oxidative phosphorylation, may further modify B-cell function and contribute to treatment heterogeneity in children (19).

In female patients, sex hormones play a pivotal role in shaping B-cell biology. Estrogen promotes B-cell survival, differentiation, and antibody production, increasing the likelihood of autoreactive B-cell expansion and heightened immune activation (21). Clinically, female AA patients often exhibit elevated inflammatory markers and higher autoantibody tendencies, which may contribute to more severe or refractory disease phenotypes (18). Hormonal modulation of T regulatory cells (Tregs) and Bregs may also exacerbate immune imbalance and facilitate the persistence of autoimmune responses.

**Figure 1:**
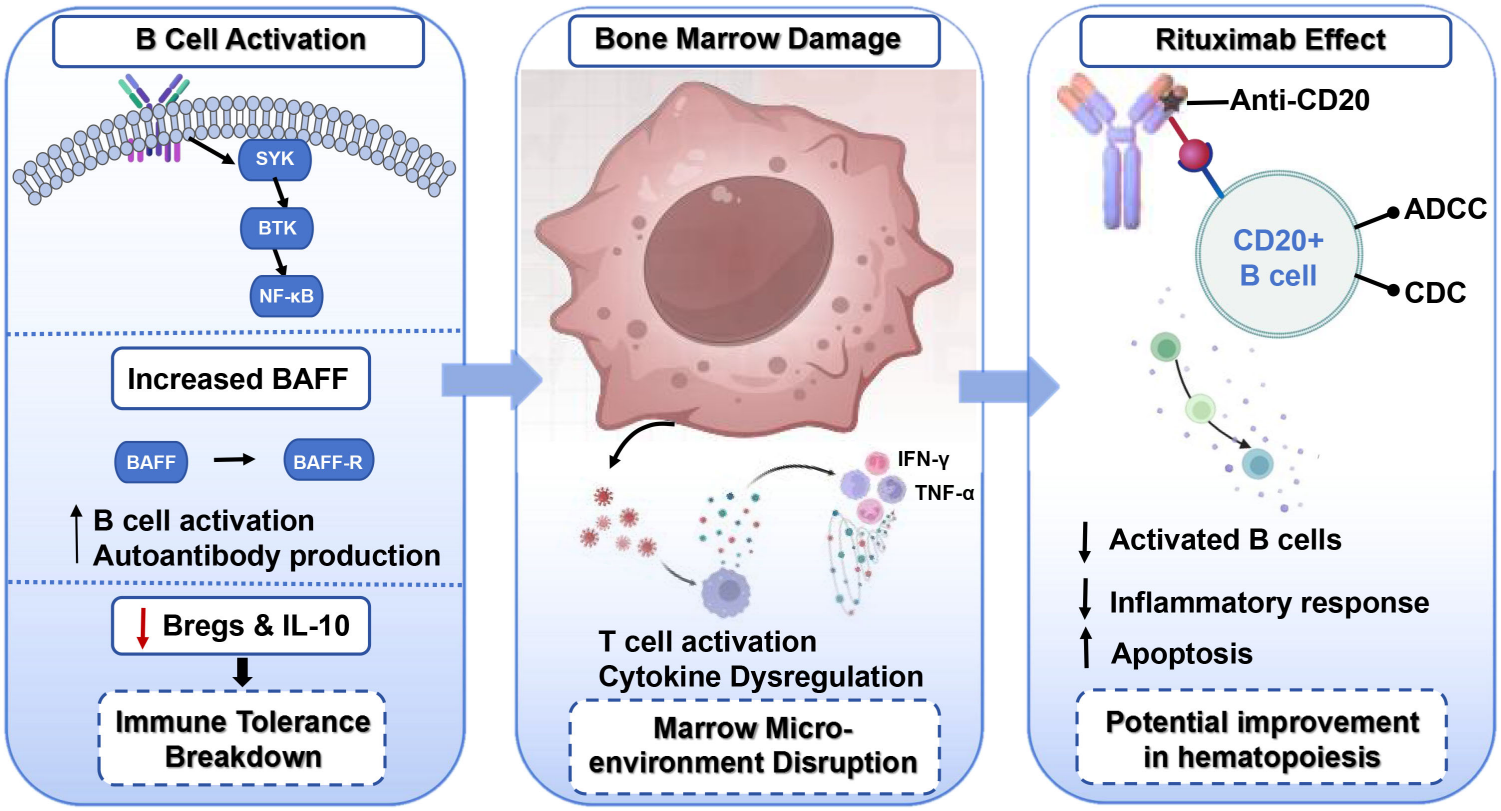
Proposed mechanisms linking B-cell dysregulation, bone-marrow injury, and the immunologic effects of rituximab in acquired aplastic anemia (AA).

Together, these findings highlight meaningful age- and sex-associated differences in B-cell activation, phenotype, and gene-regulatory pathways in AA. Such heterogeneity underscores the importance of tailored therapeutic approaches. Pediatric patients may respond differently to immunosuppressive therapy than adults (22), while female patients may require consideration of hormonal impacts on B cell function. Individualized use of B cell-targeted therapies like rituximab could optimize outcomes in these subgroups. **Figure 1** provides an integrated schematic of these immunologic differences and their potential implications for AA pathogenesis and therapeutic design, reinforcing the relevance of population-specific immune signatures for future targeted interventions.

## 3 The role and mechanism of rituximab in AA treatment

### 3.1 Pharmacological effects of rituximab

Rituximab is a chimeric monoclonal antibody specifically targeting the CD20 molecule present on the surface of mature B cells, leading to their depletion through well-established mechanisms including antibody-dependent cell-mediated cytotoxicity (ADCC) and complement-dependent cytotoxicity (CDC). In ADCC, rituximab-opsonized B cells are eliminated through interactions with Fcγ receptor–bearing effector cells such as natural killer cells, whereas in CDC, complement activation culminates in membrane attack complex formation and B-cell lysis. These mechanisms form the biological basis for rituximab use across a variety of antibody-mediated immune disorders.

In the context of autoimmune-related AA, emerging evidence suggests that aberrantly activated B cells may contribute to hematopoietic suppression, partly through the production of autoantibodies directed against hematopoietic elements. However, direct mechanistic studies confirming rituximab’s immunological effects specifically in AA remain limited, and much of the current understanding derives from analogy with other autoimmune cytopenias rather than AA-focused experimentation.

In addition to direct B cell clearance, rituximab can modulate the broader immune milieu by indirectly reducing T-cell activation and promoting the recovery of immunoregulatory populations such as Tregs and Bregs (23, 24). These downstream effects have been documented in multiple autoimmune diseases and provide a plausible mechanistic rationale—though not yet AA-validated—for how rituximab might contribute to immune tolerance restoration in selected AA patients. Consequently, while rituximab offers biologically coherent strategies for targeting B-cell–mediated autoimmunity, its mechanistic effects in AA should currently be regarded as hypothesis-generating rather than established therapeutic pathways.

### 3.2 Clinical application of rituximab in pediatric AA patients

In pediatric aplastic anemia, rituximab has been explored as an adjunctive or salvage immunomodulatory option, although its use remains investigational and supported only by limited evidence. Importantly, rituximab’s more established role in pediatrics derives from its effectiveness in immune-mediated cytopenias (IMCs) after HSCT, such as autoimmune hemolytic anemia, immune thrombocytopenia, and immune neutropenia. As highlighted by Spadea et al. (25), IMCs represent immune reconstitution disorders driven predominantly by aberrant B- and T-cell activation, for which rituximab is frequently effective and widely utilized. These post-HSCT IMCs should be clearly distinguished from relapse or progression of SAA, which reflects renewed hematopoietic stem-cell failure rather than antibody-mediated cytopenia and therefore requires fundamentally different management. Consequently, clinical experience with rituximab in IMCs cannot be directly extrapolated to SAA, where evidence remains sparse.

Within AA itself, small case series suggest that selected pediatric patients may show qualitative hematologic improvement following rituximab therapy, including rising blood counts and reduced transfusion dependence (26, 27). These outcomes, however, are modest compared with standard first-line immunosuppressive therapy (IST), in which hATG plus cyclosporine typically achieves 60–70% overall response rates, and eltrombopag-augmented IST can exceed 70–80% in pediatric cohorts. Thus, rituximab should not be viewed as comparable to or substitutive for guideline-endorsed IST.

Optimization of rituximab dosing regimens is another emerging area of interest. Notably, single-dose rituximab regimens in pediatric immune cytopenias have demonstrated comparable efficacy to multi-dose protocols, with reduced treatment burden, lower cost, and similar safety profiles (28). Such findings provide a rationale for evaluating low-dose or abbreviated rituximab regimens in pediatric AA.

In addition, monitoring anti-rituximab antibodies (ARAs) and CD20^+^ B-cell repopulation kinetics may help assess relapse risk and support individualized treatment tailoring (29). Emerging therapeutic strategies also consider combining rituximab with next-generation B-cell-targeted agents, such as anti-CD19 antibody-drug conjugates or B-cell signaling inhibitors, which may further enhance depletion depth and durability (30). Although pediatric patients generally exhibit faster immune reconstitution than adults, the long-term implications of rituximab on immune development in AA remain undefined.

Taken together, rituximab’s role in pediatric AA should be regarded as exploratory, off-label, and limited to selected refractory or immune-dominant cases, distinctly separate from its well-established application in post-HSCT immune cytopenias.

### 3.3 Therapeutic effects of rituximab in female AA patients

Interest in potential sex-specific differences in rituximab response among female AA patients has largely arisen from mechanistic observations in other autoimmune disorders rather than from AA-specific clinical evidence. While estrogen is known to influence B-cell development, survival, and antibody production, thereby providing a biologically plausible framework for sex-modulated immune responses (21), no prospective or sex-stratified clinical studies in AA have demonstrated differential responses to rituximab, and the hormone–B-cell interplay remains untested in the AA context. As a result, the notion that females may experience a more favorable clinical response should be regarded as a hypothesis rather than an established clinical pattern.

Experimental studies in autoimmune models show that B-cell depletion can reduce autoantibody levels and inflammatory mediators, and some clinical observations suggest that females may exhibit faster B-cell reconstitution after rituximab therapy (31–33). However, these findings derive from rheumatologic, neuroimmune, or renal autoimmune conditions, and their applicability to AA—which has distinct immunopathologic features—remains unproven. Available AA-specific studies are too limited to determine whether treatment responses differ between females and males, as no sex-stratified analyses have been reported in major trials or guidelines. By contrast, robust evidence supports the efficacy of standard IST, which achieves 60–70% response rates, and eltrombopag-augmented IST regimens frequently exceed 70–80%.

As in pediatric AA, rituximab has been explored in females primarily in refractory or immune-dominant disease, often in combination with conventional IST. Such regimens may deepen immunomodulation by targeting both T- and B-cell compartments (28, 34), but supporting evidence remains limited to small off-guideline case series. Additional considerations—including dosing strategies, monitoring of B-cell repopulation, and detection of ARAs—may assist individualized decision-making, though these approaches have not been systematically validated in AA (29). Emerging B-cell–directed therapeutics, such as anti-CD19 antibody-drug conjugates or signaling inhibitors, may offer mechanistic advantages, but their role in sex-specific treatment paradigms is entirely speculative (30).

Overall, rituximab should be regarded as an exploratory, non-standard option for a small subset of female patients with refractory or immune-active AA, and its clinical role remains unproven. Rigorous AA-specific, sex-stratified studies are needed to clarify true response rates, define optimal treatment timing, and identify biological markers that may predict benefit.

### 3.4 Immune reconstitution and potential tolerance pathways after rituximab therapy

Post-rituximab immune reconstitution plays a decisive role in long-term treatment success and relapse prevention. Recovery of B cell subsets, particularly Bregs, is associated with re-establishment of immune tolerance and sustained hematological remission (10, 18). Early recovery of Bregs and normalization of B-cell activation markers such as CD86 and HLA-DR may serve as indicators of improved immune homeostasis. Likewise, transcriptional regulators including BLIMP-1, which governs B-cell differentiation and regulatory function, have been proposed as potential biomarkers of immune normalization following rituximab therapy (18).

Accumulating clinical experience indicates that rituximab may improve long-term outcomes in selected pediatric and female AA patients. Data from pediatric neuroimmune and hematologic diseases demonstrate that early incorporation of rituximab can shorten steroid exposure, reduce cumulative immunosuppression, and increase complete remission rates (34, 35). These findings, although derived from related immune-mediated conditions rather than AA alone, support the broader concept that B-cell modulation may facilitate more favorable long-term immune reconstitution.

Despite these potential benefits, vigilance is required to monitor infections and delayed immune recovery, especially in patients receiving rituximab in combination with antithymocyte globulin or cyclosporine. Proactive infection prophylaxis, immunoglobulin monitoring, and individualized tapering of immunosuppression are essential to sustain therapeutic gains and minimize complications. A careful balance between sufficient immunosuppression to control autoimmunity and adequate immune restoration to avoid infection is critical for optimizing prognosis.

Taken together, these observations underscore that immune reconstitution dynamics—particularly the restoration of Bregs and normalization of B-cell and transcriptional markers—are central determinants of long-term outcomes after rituximab therapy. Continued longitudinal monitoring and integration of immune biomarkers may guide personalized immunotherapy strategies and improve durability of remission in selected AA populations. A summary of rituximab-related clinical and experimental evidence supporting these observations is provided in **Table 1**.

**Table 1:** Summary of Rituximab-related studies relevant to aplastic anemia context.

Study	Population	AA Subtype relevance	Rituximab regimen	Cohort size	Response rate	Study design
Zhang F et al., 2024 (27)	Pediatric EBV-associated lymphoproliferative disease after aplastic anemia with haplo-identical HSCT	Post-HSCT immune cytopenia	Standard rituximab (375 mg/m² weekly × 1–4 doses)	n = 30	EBV viral clearance achieved	Prospective single-center
Dong J et al., 2024 (26)	Pediatric aggressive B-cell lymphoma/leukemia	Mechanistic relevance to B-cell depletion	Rituximab + chemotherapy	n = 85	~70% ORR in lymphoma	Prospective
Baykal et al., 2025 (39)	Pediatric autoimmune rheumatic diseases	Relevant for immune reconstitution kinetics	Variable rituximab regimens	n = 27	Disease-specific	Retrospective
Zhu XJ et al., 2022 (28)	Pediatric primary immune thrombocytopenia (ITP)	Immune cytopenia; partial analogy to AA	100 mg/m² × 1 dose vs. 375 mg/m² × 4 doses	n = 90	~50–60% ORR in ITP	Prospective, comparative
Tong YN et al., 2024 (34)	Pediatric myasthenia gravis	Autoimmune model relevant to B-cell depletion effects	Rituximab (375 mg/m² weekly × 4)	n = 27	78% clinical improvement	Retrospective
Wang JJ et al., 2025 (29)	Frequently relapsing or steroid-dependent nephrotic syndrome (FR/SDNS)	B-cell recovery and relapse risk relevant for AA rituximab monitoring	Rituximab (375 mg/m² × 1–2 doses)	n = 48	Relapse reduction rate significant	Prospective
Gao HX et al., 2024 (30)	Pediatric Hodgkin lymphoma (immune-targeting + chemotherapy)	B-cell depletion and immune reconstitution relevant for AA therapy design	Rituximab-containing regimen	n = 28	High remission rate (>80%)	Retrospective
Campbell N et al., 2024 (31)	Animal model of autoantibody-mediated disease	Mechanistic insight into B-cell depletion	Rituximab (250 μg/kg per day)	Animal	↓autoantibody levels; improved phenotype	Experimental
Reeve KE et al., 2022 (33)	Preeclampsia autoimmune model	Mechanistic insight	Rituximab + Anti-CD40	Animal	↓AT1-AA antibodies; ↓inflammation	Experimental

### 3.5 Safety and preventive care during rituximab therapy

Given the prolonged B-cell depletion induced by rituximab and its associated infection risks, safety monitoring and preventive measures are essential. Current guideline-level recommendations emphasize comprehensive screening and infection-prevention strategies prior to initiating B-cell–depleting therapy (36).

All patients should undergo hepatitis B virus (HBV) screening, including HBsAg, anti-HBc, and HBV DNA testing, because rituximab significantly increases the risk of HBV reactivation. Patients with evidence of current or past infection should begin antiviral prophylaxis and receive close hepatology follow-up. Immunoglobulin monitoring is recommended throughout therapy, as rituximab-associated hypogammaglobulinemia may predispose patients to severe infections; intravenous immunoglobulin replacement can be considered in symptomatic or high-risk individuals.

Vaccination management is also essential. Live vaccines should be avoided during and shortly after rituximab administration, and inactivated vaccines may exhibit limited immunogenicity. Revaccination should be considered only after adequate B-cell reconstitution, consistent with IDSA and CDC immunization guidelines for immunocompromised patients. When rituximab is administered alongside antithymocyte globulin or cyclosporine as part of combined immunosuppression, prophylaxis against *Pneumocystis jirovecii* pneumonia (PJP) is recommended according to IDSA standards.

Routine clinical surveillance, complete blood counts, and infection monitoring (including EBV or CMV in high-risk settings) should be implemented throughout therapy, particularly during periods of profound lymphopenia or delayed immune reconstitution. These preventive and monitoring strategies are critical for ensuring the safe and appropriate use of rituximab in selected cases of refractory or immune-dominant AA.

### 3.6 Guidelines and recommendations

Current international guidelines consistently emphasize that rituximab is not part of standard therapy for acquired AA. According to the BSH 2024 Adult AA Guideline (36), standard first-line treatment for adults remains horse antithymocyte globulin (hATG) combined with cyclosporine (CsA), with eltrombopag incorporated based on disease severity and transplant eligibility. Rituximab is not recommended as first-line therapy, nor is it listed as a routine second-line option in treatment algorithms. Similarly, the EWOG-SAA 2023 Consensus for Pediatric Severe Aplastic Anemia identifies matched sibling donor hematopoietic stem-cell transplantation (HSCT) or hATG+CsA-based immunosuppressive therapy as the pediatric standard of care, again excluding rituximab from frontline or standard salvage regimens (37). This consensus stresses that rituximab may only be considered in exceptional, individualized refractory cases, and its use is regarded as off-label. The ASH guidance for AA management further outlines the decision-making framework for second-line therapy following IST failure but does not highlight rituximab as a recommended option for severe AA; instead, HSCT, eltrombopag-based regimens, or repeat IST are favored (38).

Together, these guidelines demonstrate that rituximab’s role in AA remains exploratory and is supported only by small retrospective series or anecdotal reports. Accordingly, any potential use of rituximab should be restricted to selected refractory or immune-dominant AA cases and should not be interpreted as guideline-endorsed practice. This context is crucial when discussing combination strategies involving rituximab, which fall outside established treatment pathways and should be interpreted cautiously and distinguished clearly from evidence in immune cytopenias such as ITP or AIHA, where rituximab is widely utilized.

## 4 Knowledge gaps and future directions

### 4.1 Gaps in current understanding of B-cell pathogenesis in AA

Despite growing evidence that B cells participate in the immune-mediated destruction of HSPCs, major gaps remain in defining their precise mechanistic roles in acquired aplastic anemia. Although studies have demonstrated aberrant B-cell activation, altered subset distributions, and abnormal transcriptional programs—including increased CD86 and HLA-DR expression, reduced BLIMP-1 expression, and elevated frequencies of autoreactive B-cell populations—the causal contribution of these abnormalities to marrow failure is still unclear (10, 18, 20). Current data are largely descriptive and often extrapolated from related immune cytopenias, underscoring the need for functional studies that determine how specific B-cell subsets, cytokine secretion patterns, and autoantibody-producing pathways contribute to HSPC apoptosis and immune dysregulation in AA. Future research employing single-cell multi-omics, high-dimensional cytometry, and spatial immunology will be essential to map the interactions between activated B cells and the bone marrow microenvironment, thereby clarifying how B-cell–driven mechanisms integrate into the broader immunopathology of AA.

### 4.2 Need for age- and sex-stratified immunologic research

Although pediatric and female AA patients exhibit distinctive B-cell phenotypes—such as lower frequencies of transitional and regulatory B cells in children, metabolic alterations affecting immune-cell function in pediatric populations, and estrogen-enhanced B-cell activation and survival signals in females—these observations arise primarily from small cohorts or mechanistic studies in related autoimmune disorders rather than AA-specific investigations (10, 19, 21). Consequently, the true extent to which age-dependent immune maturation, hormone-modulated B-cell activity, and sex-specific patterns of autoimmunity influence AA pathogenesis, immune phenotype, or response to immunosuppressive therapy, including rituximab, remains insufficiently defined.

Furthermore, preliminary observations that pediatric patients may reconstitute B-cell compartments more rapidly after therapy or that female patients may achieve more balanced immune recovery and hematologic improvement require validation in adequately powered AA-specific studies (33, 39). Much of the evidence suggesting these differences derives from fields such as autoimmune cytopenias, nephrotic syndrome, or neuroimmune disorders—contexts in which B-cell dysregulation is better characterized but not directly translatable to AA. Distinguishing true AA-specific immunologic patterns from extrapolated mechanisms is therefore essential.

In contrast, virtually no studies have evaluated rituximab responses or B-cell immunophenotypes specifically in adult or male AA patients, and existing reports seldom stratify outcomes by age or sex. This absence of adult- and male-focused data represents a major knowledge gap and complicates interpretation of whether observed pediatric or female-associated patterns reflect biological reality or sampling bias. Without sex- and age-stratified analyses, it is not possible to determine whether B-cell–directed therapies such as rituximab exert differential effects across demographic subgroups or whether hormonal and developmental influences meaningfully alter immune reconstitution.

Together, these gaps underscore the need for comprehensive immunophenotyping and longitudinal immune-recovery studies intentionally designed to evaluate pediatric, adult, male, and female AA patients separately. Such efforts will be critical for establishing robust age- and sex-stratified biomarkers, avoiding over-reliance on extrapolated findings, and developing biologically informed strategies to guide individualized immunotherapy in AA.

### 4.3 Therapeutic limitations of CD20 targeting and future b-cell–directed strategies

Although rituximab has been explored as a salvage or adjunctive option in selected AA populations—particularly in pediatric and female patients—its use remains off-label and is supported only by small case series or extrapolations from related immune cytopenias (28–30, 34). Several biologic and clinical factors likely contribute to the limited and inconsistent efficacy of CD20-directed therapy in AA. First, CD20 is expressed on mature B cells but not on early B-cell progenitors or plasma cells; thus, rituximab may incompletely target the full spectrum of autoreactive or cytokine-producing B-cell subsets potentially involved in AA pathogenesis. Second, AA remains a predominantly T-cell–driven disorder, and B-cell depletion alone may be insufficient to suppress cytotoxic T-cell–mediated destruction of hematopoietic stem and progenitor cells. Third, pathogenic B cells in AA may reside within specialized marrow niches that limit exposure to circulating monoclonal antibodies, further reducing the depth of depletion achieved by anti-CD20 therapy.

These mechanistic limitations underscore the need for therapeutic strategies that extend beyond CD20-directed depletion to target additional pathways implicated in aberrant B-cell activation and survival. Dysregulated BCR signaling and elevated BTK activity in AA immune cells (12, 14) suggest that BTK inhibitors may offer broader modulation of B-cell activation than CD20-directed agents alone. Similarly, increased BAFF levels in immune-active marrow failure syndromes indicate that BAFF-blocking therapies could reduce survival cues for autoreactive B cells (17). Next-generation approaches—including anti-CD19 antibody–drug conjugates, CD22-targeted therapies, and small-molecule inhibitors of B-cell activation—may achieve deeper and more comprehensive modulation of the B-cell compartment than rituximab.

Finally, rational combination strategies integrating B-cell–directed agents with T-cell–targeting therapies, eltrombopag, or metabolic pathway regulators may better address the multifactorial immune dysregulation underlying AA. Such mechanism-guided therapeutic development offers a promising avenue for refractory or high-risk AA, but requires validation through prospective trials specifically designed for this disease.

### 4.4 Biomarkers and immune-monitoring frameworks

A major unmet need in AA management is the development of reliable biomarkers that can predict responsiveness to B-cell–targeted therapies and guide individualized immunosuppression. Although several candidate markers have been proposed—such as B-cell activation molecules including CD86 and HLA-DR (20), transcriptional regulators such as BLIMP-1 that reflect impaired regulatory B-cell differentiation (18), and reductions in Breg frequencies associated with immune tolerance breakdown (10, 13)—none has been validated prospectively in AA or shown to predict clinical response to rituximab. Moreover, many of these markers are derived from small cohorts or extrapolated from immune cytopenias and other autoimmune disorders, limiting their applicability to AA.

In the context of rituximab therapy, monitoring ARAs and assessing CD20^+^ B-cell repopulation kinetics may provide insights into relapse risk or incomplete immune reconstitution; however, these approaches remain investigational in AA and lack standardized thresholds or clinical decision algorithms (29). Additional challenges include variability in assay platforms, differences in pediatric versus adult immunologic baselines, and the absence of harmonized reference ranges, all of which complicate the integration of immune-monitoring markers into clinical practice.

To advance precision immunotherapy in AA, future studies should establish longitudinal immune-monitoring frameworks that incorporate cellular (Breg frequencies, activated B-cell subsets), transcriptional (BLIMP-1–related programs), and functional readouts, while linking these measures to clinical outcomes in well-defined patient subgroups. Such standardized, prospectively validated biomarker strategies will be essential for optimizing rituximab dosing schedules, anticipating immune-reconstitution complications, and minimizing infection risks during prolonged immune suppression.

### 4.5 Priorities for future clinical trials

Future clinical trials investigating B-cell-directed therapies in AA should incorporate biomarker-guided designs, age- and sex-specific recruitment, and rigorous assessment of immune-reconstitution endpoints. Multicenter randomized controlled trials are needed to clarify the therapeutic role of rituximab as an adjunct or salvage option, particularly given that current evidence derives primarily from retrospective studies and related autoimmune cytopenias (28–30, 34, 35). Biomarker-enriched enrollment strategies—such as selecting patients with high BAFF levels, heightened B-cell activation signatures, or reduced Breg frequencies—may improve the likelihood of identifying subgroups most likely to benefit from B-cell-targeted interventions (10, 17, 20). Additionally, prospective tracking of immune-reconstitution dynamics, infection rates, and long-term hematologic stability should be integrated as core trial endpoints. Such rigorously designed studies will be crucial for defining the place of rituximab and other B-cell-directed therapies in AA treatment algorithms and for advancing precision immunotherapy tailored to patient-specific immunologic profiles.

### 4.6 Limitations

This review has several limitations that should be acknowledged. First, the available evidence on rituximab in acquired AA is extremely limited, consisting primarily of case reports, small retrospective series, or extrapolations from immune-mediated cytopenias and other autoimmune disorders. As a result, many observations regarding B-cell activation, immune reconstitution, and potential rituximab responsiveness are hypothesis-generating rather than definitive. Second, although we summarized emerging age- and sex-related immunologic patterns, AA-specific, stratified datasets remain sparse, and much of the existing literature derives from pediatric immune cytopenias, nephrotic syndrome, rheumatologic disease, or neuroimmune conditions. These contextual data may not fully reflect AA biology and carry a risk of over-extrapolation. Third, despite implementing a structured literature search, publication bias is likely, given the preferential reporting of rituximab-responsive cases and the absence of prospective negative studies. Fourth, the heterogeneity of definitions across studies—such as varying diagnostic criteria, response assessment methods, and treatment regimens—limits direct comparability and complicates interpretation of rituximab’s potential role in AA. Finally, no randomized controlled trials have evaluated anti-CD20 therapy in AA, and biomarker candidates discussed in this review have not been prospectively validated. Therefore, conclusions regarding rituximab’s utility, particularly in pediatric or female patients, should be interpreted cautiously and regarded as exploratory pending rigorous AA-specific clinical trials.

## 5 Conclusion

Aplastic anemia is a complex immune-mediated marrow failure syndrome in which aberrant B-cell activation contributes to immune dysregulation, although its precise pathogenic role remains insufficiently defined. Rituximab and other B-cell–directed approaches offer biologically plausible but still exploratory therapeutic avenues, supported mainly by small retrospective studies and extrapolated evidence rather than AA-specific prospective trials; accordingly, anti-CD20 therapy should not be considered part of standard AA management. Observations suggesting potential age- or sex-associated differences in immune phenotype or treatment response—particularly in pediatric and female patients—remain hypothesis-generating and require validation in rigorously designed, stratified cohorts. Future progress will depend on clarifying B-cell–mediated mechanisms of marrow injury, developing reliable biomarkers to guide individualized immunotherapy, and conducting multicenter, biomarker-enriched clinical trials to define the appropriate role of rituximab and emerging B-cell–targeted strategies. Through integration of mechanistic and clinical research, more precise and effective treatment paradigms for acquired aplastic anemia may ultimately be achieved.
